# Smoking prevalence and trends among a U.S. national sample of women of reproductive age in rural versus urban settings

**DOI:** 10.1371/journal.pone.0207818

**Published:** 2018-11-28

**Authors:** Tyler D. Nighbor, Nathan J. Doogan, Megan E. Roberts, Antonio Cepeda-Benito, Allison N. Kurti, Jeff S. Priest, Harley K. Johnson, Alexa A. Lopez, Cassandra A. Stanton, Diann E. Gaalema, Ryan Redner, Maria A. Parker, Diana R. Keith, Amanda J. Quisenberry, Stephen T. Higgins

**Affiliations:** 1 Vermont Center on Behavior and Health, University of Vermont, Burlington, Vermont, United States of America; 2 Department of Psychiatry, University of Vermont, Burlington, Vermont, United States of America; 3 Ohio Colleges of Medicine Government Resource Center, The Ohio State University, Columbus, OH, United States of America; 4 Department of Psychological Science, University of Vermont, Burlington, Vermont, United States of America; 5 Medical Biostatistics, University of Vermont, Burlington, Vermont, United States of America; 6 Westat, Center for Evaluation and Coordination of Training and Research (CECTR) in Tobacco Regulatory Science, Rockville, Maryland, United States of America; 7 Department of Oncology, Georgetown University Medical Center, Washington District of Columbia, United States of America; 8 Rehabilitation Institute, Southern Illinois University, Carbondale, Illinois, United States of America; 9 Department of Health Behavior, Roswell Park Comprehensive Cancer Center, Buffalo, New York, United States of America; Brown University, UNITED STATES

## Abstract

U.S. smoking prevalence is declining at a slower rate in rural than urban settings and contributing to regional health disparities. Cigarette smoking among women of reproductive age is particularly concerning due to the potential for serious maternal and infant adverse health effects should a smoker become pregnant. The aim of the present study was to examine whether this rural-urban disparity impacts women of reproductive age (ages 15–44) including pregnant women. Data came from the ten most recent years of the U.S. National Survey on Drug Use and Health (2007–2016). We estimated prevalence of current smoking and nicotine dependence among women categorized by rural-urban residence, pregnancy status, and trends using chi-square testing and multivariable modeling while adjusting for common risk factors for smoking. Despite overall decreasing trends in smoking prevalence, prevalence was higher among rural than urban women of reproductive age overall (*χ*^*2*^(1) = 579.33, *p* < .0001) and among non-pregnant (*χ*^*2*^(1) = 578.0, *p* < .0001) and pregnant (*χ*^*2*^(1) = 79.69, *p* < .0001) women examined separately. An interaction between residence and pregnancy status showed adjusted odds of smoking among urban pregnant compared to non-pregnant women (AOR = .58, [.53 –.63]) were lower than those among rural pregnant compared to non-pregnant women (AOR = 0.75, [.62 –.92]), consistent with greater pregnancy-related smoking cessation among urban pregnant women. Prevalence of nicotine dependence was also higher in rural than urban smokers overall (*χ*^*2*^(2) = 790.42, *p* < .0001) and among non-pregnant (*χ*^2^(2) = 790.58, *p* < .0001) and pregnant women examined separately (*χ*^2^(2) = 63.69, *p* < .0001), with no significant changes over time. Associations involving residence and pregnancy status remained significant in models adjusting for covariates (*p*s < 0.05). Results document greater prevalence of smoking and nicotine dependence and suggest less pregnancy-related quitting among rural compared to urban women, disparities that have potential for direct, multi-generational adverse health impacts.

## Introduction

Rural communities in the United States (U.S.) are increasingly characterized by socioeconomic and health disparities [1–4]. Rural areas often have a higher prevalence of health-related risk behaviors, including the highest cigarette smoking rates in the country [5–10]. Additionally, individuals residing in rural settings often have poorer cessation-related outcomes than those living in urban areas [10]. Compared to non-rural (urban and suburban, hereafter referred to as urban) areas in the U.S., rural areas have overall higher mortality rates [11–13] particularly as related to smoking-related diseases including ischemic heart disease, obstructive pulmonary disease, and lung cancer [11–13].

Although overall smoking prevalence is decreasing in the U.S. [14–15] this decline is occurring at a lower rate in rural than urban settings [5–6, 15]. Within rural communities, sex appears to contribute to growing disparities in smoking prevalence, with rural-urban differences being more pronounced among females than males and not fully accounted for by socio-demographic risk factors for smoking [5]. Smoking among women of reproductive age (ages 15–44) is of particular concern due to adverse impacts on mother and infant health should these women become pregnant, as well as the risks of second-hand smoke exposure should they be parenting young children [16–17]. We know of no reports comparing smoking among rural versus urban women of reproductive age. A conference presentation by investigators from the American Lung Association reviewed prevalence data among pregnant and non-pregnant women of reproductive age using the 2009 National Survey on Drug Use and Health (NSDUH) noting that (a) overall crude smoking rates were greater among rural than urban women and (b) while pregnant urban women smoked less than their non-pregnant counterparts, there was no significant difference between rural pregnant compared to non-pregnant women, suggesting that there was less pregnancy-related quitting among rural versus urban women [15]. Hence, the overarching purpose of the present study was to examine rural versus urban differences in smoking prevalence among women of reproductive age and whether rural women may be more likely to continue smoking during pregnancy.

We are unaware of any prior reports on whether prevalence of nicotine dependence differs between rural versus urban smokers, but of course nicotine dependence is a major contributor to the emergence and persistence of chronic smoking [18–19] and is a robust predictor of difficulties in quitting cigarette smoking in clinical samples and self-quitters [20–23]. Thus, comparing prevalence of nicotine dependence between rural versus urban women of reproductive age who smoke was a secondary aim of the present study.

## Materials and methods

### Data source

Data were obtained from the National Survey on Drug Use and Health (NSDUH), an annual, multi-year U.S. nationally representative cross-sectional survey. Detailed descriptions of survey procedures have been provided previously for each of the survey years [24]. We used the ten most recent years (2007–2016) at the time the study was conducted. Data were analyzed based on consecutive two-year periods (e.g., 2007 and 2008; 2009 and 2010) due to the relatively smaller number of rural pregnant women. Participant weights were included with the survey data to obtain results representative of the U.S. population by correcting for selection probabilities, non-response, and post-stratification. Hereafter, references to “adjusted” or “unadjusted” models refer to covariate adjustment, rather than to weight adjustments for representativeness.

### Measures

The first dependent variable, “current smoking,” was defined as self-reported use of at least one cigarette in the past 30 days and at least 100 lifetime cigarettes. “Nicotine dependence” served as the second dependent variable and was defined as how soon after waking the participant smoked their first cigarette, the first item on the Fagerstrom Test for Nicotine Dependence (FTND; [25]). Time to first cigarette after waking is a robust predictor of success in quitting smoking [26–27]. Those who reported smoking within 30 min were classified as nicotine dependent. The key predictor variables included pregnancy status and geographic setting (urban non-pregnant women of reproductive age, urban pregnant women, rural non-pregnant women of reproductive age, and rural pregnant women), and the interactions between pregnancy status and geographic setting. Geographic setting was a county level classification from the Office of Management and Budget called the Rural Urban Continuum Codes (based on the 2000 Census data and 2013 statistical area classifications). As in previous studies [5–6, 15], we classified respondents as urban if they were from core counties that are part of an urbanized area with a population size > 10,000, or an outlying county with 25% or more of its labor force tied to a core county by commuting flows. Rural participants were those not residing in an urban area.

The adjusted model included covariates that are known risk factors for smoking [5–6, 28] including five categorical variables (age, race, education, marital status, and income), and four dichotomous variables (unemployed, past year major depressive episode, health insurance (any type), and past year substance abuse).

### Statistical analyses

Ten years of NSDUH data were combined (2007–2016) (*n* = 561,231) and the person-level sample weight was divided by 10. Across all analyses, strata, cluster, and weight were included. We calculated frequencies using PROC SURVEYFREQ in SAS 9.4 (SAS Institute, Cary, NC), relying on Rao-Scott chi-square tests to compare current smoking status and time to first cigarette after waking by geographic setting, sex, pregnancy status, age, and time. The sample was then limited to females of reproductive age only (*n* = 199,486), and multiple logistic regression procedures were used to model current smoking status and time to first cigarette after waking (PROC SURVEYLOGISTIC in SAS 9.4). Purposeful selection of covariates [29] was used to build models, starting with a univariable analysis of all prospective covariates. Any variable whose univariable test had a *p*-value of .25 or less was included in an initial multivariable model. Variables not contributing significantly to predicting the outcome at a *p*-value less than .05 were winnowed out of multivariable models until only significant contributors remained [29]. Initially excluded or dropped variables were checked again, one-by-one, in multivariable models containing only significant predictors. The interaction between urban/rural status and pregnancy status was added to a tentatively final multivariable model and retained if it contributed significantly to the outcome. Final logistic models included area under the Receiver Operating Characteristic (ROC) curves to evaluate sensitivity and specificity. Across all analyses, statistical significance was defined as *p* < .05 (2-tailed).

## Results

### Study sample

The analytic sample included 199,486 female participants who responded to all necessary survey items. Rural and urban populations included 40,166 (20.2%) and 158,250 (79.8%) respondents, respectively. Regarding geographic residence by pregnancy status, the sample included 38,228 (19.3%) rural non-pregnant women, 1,938 (0.1%) rural pregnant women, 151,826 (76.5%) urban non-pregnant women, and 6,424 (3.2%) urban pregnant women. A total of 40,308 (20.8%) participants were classified as current cigarette smokers.

Table 1 shows descriptive statistics for the sample overall, and separately with women categorized by rural-urban residence and pregnancy status. Comparisons of the descriptive statistics show that between 2007–2016, rural women were less racially diverse, had lower levels of education, lower incomes, and were more likely to be current smokers. Differences in the distribution of each of the categorical variables in Table 1 were tested individually using chi-square tests for differences between urban and rural populations, and each was statistically significant (all *ps* < .05*)*.

**Table 1:** Descriptive statistics of the combined 2007–2016 sample of women of reproductive age (national survey on drug use and health).

			Non-pregnant women				Pregnant Women			
	Overall (n = 199,486)		Rural (n = 38,228)		Urban (n = 151,826)		Rural (n = 1,938)		Urban (n = 6,424)	
	Sample n	%	Sample n	%	Sample n	%	Sample n	%	Sample n	%
Age										
15–17	42,392	10.00	8,868	11.16	32,767	10.15	116	3.57	374	2.43
18–25	90,136	27.09	16,812	26.64	67,678	26.76	1,307	45.65	3,848	34.22
26–44	66,958	62.90	12,548	62.19	51,381	63.09	515	50.78	2,202	63.36
Race										
White	115,814	58.39	28,350	77.25	82,463	55.40	1,353	72.85	3,137	54.75
African American	27,698	14.02	2,837	9.53	23,307	14.69	176	13.52	1,187	14.92
Native American	3,090	0.57	1,512	1.82	1,367	0.36	120	1.91	65	0.25
Native Hawaiian/ Pacific Islander	1,069	0.43	212	0.26	801	0.47	16	0.26	34	0.37
Asian	8,379	6.10	608	1.44	7,473	6.87	23	0.88	1,524	6.92
Latina	36,406	18.84	3,370	8.26	31,066	20.53	193	8.59	1,534	21.06
Other	7,030	1.65	1,339	1.44	5,349	1.68	57	2.00	237	1.74
Education										
12-17-year olds	42,392	10.01	8,868	11.16	32,767	10.15	116	3.57	374	2.43
< High school	7,030	10.93	4,478	13.03	15,570	10.38	367	16.11	1,185	14.31
High school	45,582	22.83	9,876	28.62	32,879	21.79	690	31.71	1,865	23.40
Some college	53,038	28.50	10,173	30.20	40,467	28.39	504	29.17	1,673	25.06
≥ College	36,695	27.73	4,833	16.98	30,143	29.29	261	19.44	1,327	34.80
Income										
≤ $20,000	52,862	21.57	11,132	26.18	38,794	20.71	661	31.68	1,919	21.52
$20,000 to $49,999	66,514	31.72	13,748	36.31	49,363	31.03	743	34.02	2,298	29.62
$50,000 to $74,999	31,135	16.73	6,176	17.62	23,602	16.58	270	17.83	946	17.22
≥ $75,000	48,975	29.97	7,172	19.89	40,067	31.68	264	16.47	1,261	31.64
Unemployed	17,817	6.97	3,196	7.25	13,692	6.93	176	8.67	611	6.52
Married	51,037	40.36	10,337	42.46	36,605	39.16	875	51.42	2,948	60.36
Major Depressive Episode in past year	17,282	9.22	3,445	10.30	13,185	9.20	146	8.48	410	5.38
Health Insurance	167,014	82.60	31,457	79.39	127,291	82.81	1,718	87.47	5,726	90.90
Substance Abuse	9,318	3.39	1,643	3.03	7,152	3.42	105	4.68	318	3.53
Current Cigarette Use	40,308	20.81	9,995	29.62	28,549	19.70	489	23.09	1,014	11.73

### Rural-urban comparisons on prevalence of current smoking

#### Overall prevalence

**Fig 1:**
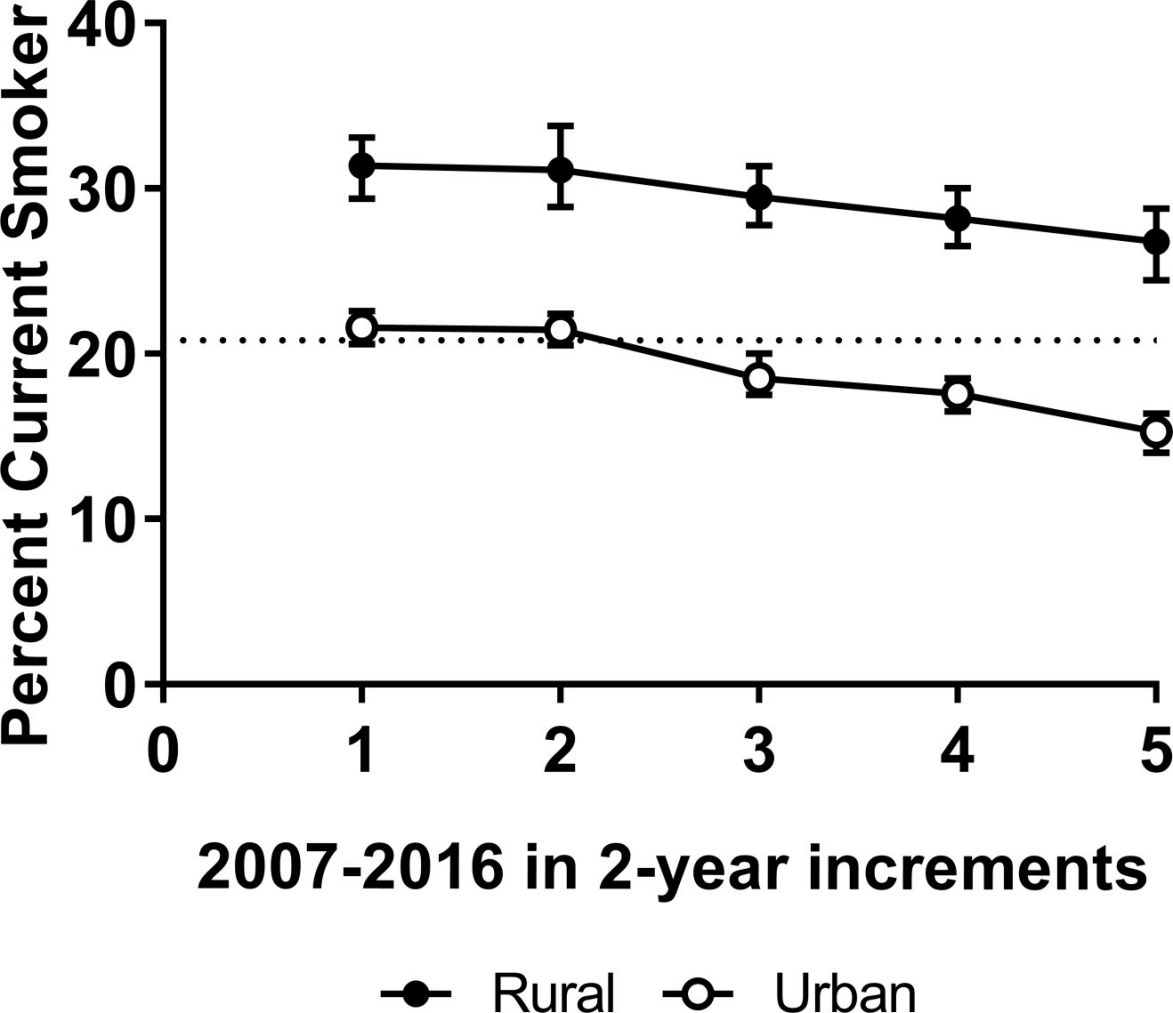
Current cigarette smoking prevalence among rural versus urban women in 2-year increments with 95% confidence bars. Estimates are weighted to reflect the U.S. population during the years 2007–2016.

Overall prevalence of current smoking for rural and urban women across the 10-year period is shown in two-year increments in Fig 1, with prevalence greater among rural than urban women throughout (*χ*^*2*^ (1) = 579.33, *p* < .0001). Significant differences were observed between rural and urban women during 2007–2008 (*χ*^*2*^ (1) = 29.44, *p* < .0001), 2009–2010 (*χ*^*2*^ (1) = 37.15, *p <* .0001), 2011–2012 (*χ*^*2*^ (1) = 80.06, *p* < .0001), 2013–2014 (*χ*^*2*^ (1) = 126.16, *p* < .0001), and 2015–2016, (*χ*^*2*^ (1) = 118.51, *p* < .0001). Prevalence decreased over time for rural (*χ*^*2*^ (4) = 20.85, *p* < .0003) and urban women (*χ*^*2*^ (4) = 203.07, *p* < .0001), although the decrease was more pronounced in urban women, with considerable overlap in the confidence intervals over time among rural women.

#### Prevalence by pregnancy status

**Fig 2:**
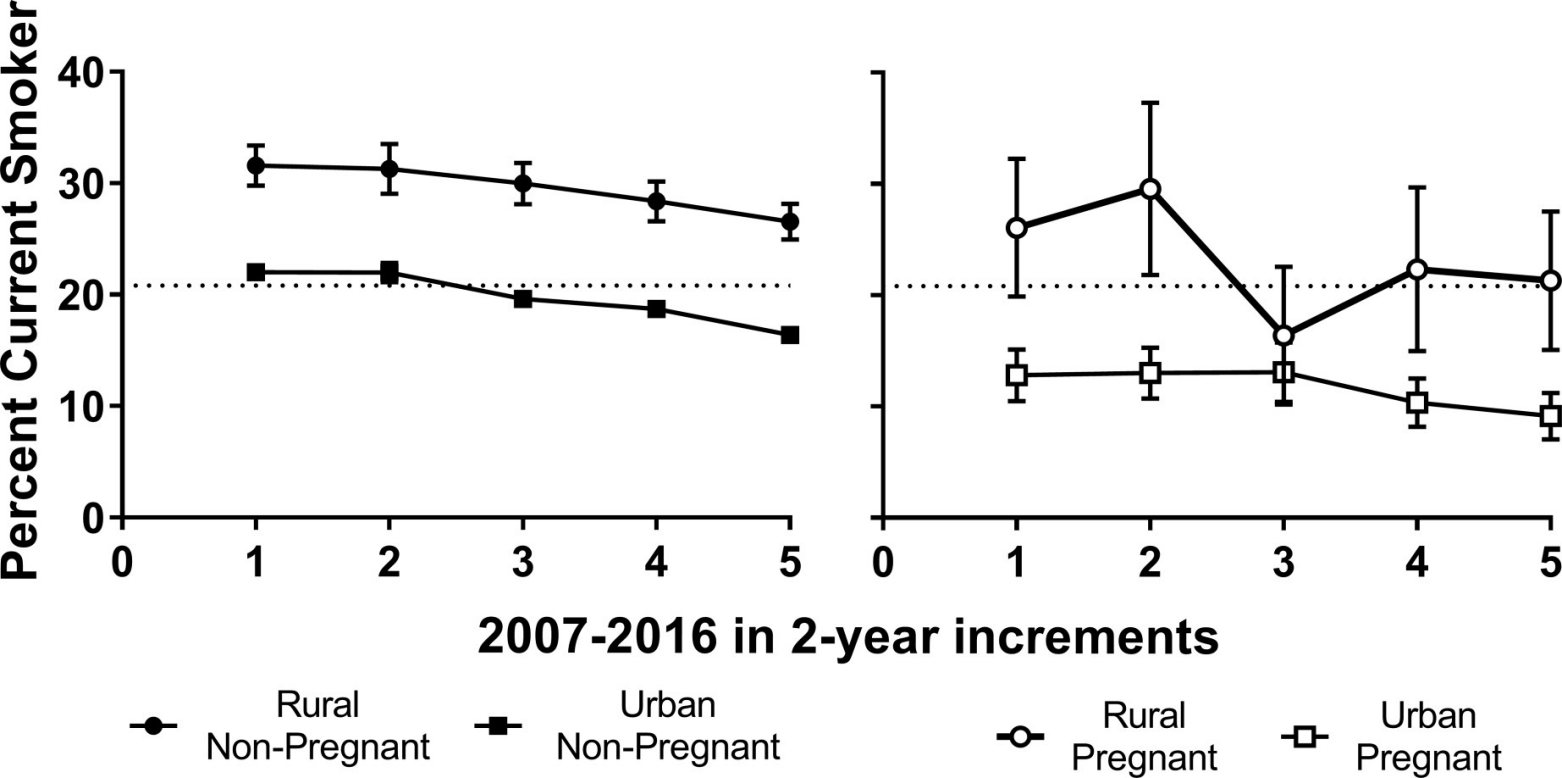
Current cigarette smoking prevalence among rural versus urban women, separated by pregnancy status, in 2-year increments with 95% confidence bars. Estimates are weighted to reflect the U.S. population during the years 2007–2016.

Prevalence of current smoking across the 10-year period for rural and urban women categorized by pregnancy status is shown in two-year increments in Fig 2. Overall prevalence of smoking across the ten-year period was higher among non-pregnant rural than urban women (*χ*^*2*^ (1) = 578.0, *p* < .0001). Prevalence of smoking among rural non-pregnant women was significantly higher than among urban non-pregnant women in 2007–2008 (*χ*^*2*^ (1) = 130.42, *p* < .0001), 2009–2010 (*χ*^*2*^ (1) = 63.22, *p <* .0001), 2011–2012 (*χ*^*2*^ (1) = 135.62, *p* < .0001), 2013–2014 (*χ*^*2*^ (1) = 144.38, *p* < .0001), and 2015–2016, (*χ*^*2*^ (1) = 204.79, *p* < .0001). Prevalence decreased over time for rural (*χ*^*2*^ (4) = 19.10, *p* = 0.001) and urban non-pregnant women (*χ*^*2*^ (4) = 195.84, *p* < .0001), but again remained higher for rural than urban women throughout.

Overall prevalence of smoking among rural pregnant women was significantly higher than urban pregnant women across the 10-year period (*χ*^*2*^ (1) = 79.69, *p* < .0001). Prevalence of smoking among rural pregnant women was significantly higher than among urban pregnant women in 2007–2008 (*χ*^*2*^ (1) = 26.04, *p* < .0001), 2009–2010 (*χ*^*2*^ (1) = 27.09, *p <* .0001), 2013–2014 (*χ*^*2*^ (1) = 16.02, *p* < .0001), and 2015–2016, (*χ*^*2*^ (1) = 17.51, *p* < .0001), although not in 2011–2012 (*χ*^*2*^ (1) = .91, *p* = .3433). Neither rural (*χ*^*2*^ (4) = 8.49, *p* = .08) nor urban pregnant women (*χ*^*2*^ (4) = 9.21, *p* = .06) showed significant decreases in smoking prevalence over time.

#### Multivariable modeling of current smoking

Table 2 shows results of the multivariable logistic regression models on prevalence of current smoking. Even after adjusting for the selected smoking risk factors, the odds of current smoking were greater for rural than urban women overall (AOR = 1.10, 95% CI [1.06–1.15]) and lower for pregnant than non-pregnant women (AOR = .61, [.56 –.67]).

**Table 2:** Adjusted odds ratios (and 95% confidence intervals) from models of current smoking in the US between the years 2007–2016.

	Adjusted Model (No Interaction)				Adjusted Model (1 Interaction)			
	OR	95% CI		*p*	OR	95% CI		*p*
Rural Residence								
Yes	1.10	1.06	1.15	**< .0001**	**—**	**—**	**—**	
No	Ref							
Pregnancy Status								
Yes	.61	.56	.67	**< .0001**	**—**	**—**	**—**	
No	Ref							
Rurality by Pregnancy Status								
Rural, Pregnant vs. Non-Pregnant	—	—	—		.75	.62	.92	**.005**
Urban, Pregnant vs. Non-Pregnant	—	—	—		.58	.53	.63	**< .0001**
Non-Pregnant, Urban vs. Rural	—	—	—		.80	.77	.85	**< .0001**
Pregnant, Urban vs. Rural	—	—	—		.62	.50	.76	**< .0001**
Time								
2007–2008	1.19	1.12	1.26	**< .0001**	1.19	1.12	1.26	**< .0001**
2009–2010	1.20	1.13	1.27	**< .0001**	1.20	1.13	1.27	**< .0001**
2011–2012	1.10	1.04	1.16	**.001**	1.10	1.04	1.16	**.001**
2013–2014	1.05	.99	1.11	.092	1.05	.99	1.11	.092
2015–2016	Ref							
Education								
12-17-year-olds	.04	.02	.08	**< .0001**	.04	.02	.08	**< .0001**
< High School	3.47	3.23	3.72	**< .0001**	3.46	3.22	3.71	**< .0001**
High School Graduate	2.88	2.71	3.07	**< .0001**	2.88	2.71	3.06	**< .0001**
Some College	2.34	2.20	2.48	**< .0001**	2.33	2.20	2.47	**< .0001**
College Graduate	Ref							
Income								
Less than $20,000	1.63	1.54	1.73	**< .0001**	1.64	1.54	1.74	**< .0001**
$20,000 to $49,999	1.36	1.30	1.43	**< .0001**	1.37	1.30	1.43	**< .0001**
$50,000 to $74,999	1.13	1.06	1.20	**.0002**	1.13	1.06	1.20	**.0002**
(≥ $75,000)	Ref							
Race/Ethnicity								
African American	.48	.45	.51	**< .001**	.48	.45	.51	**< .0001**
Native American	1.15	.96	1.37	.137	1.14	.95	1.37	.153
Native Hawaiian / Pacific Islander	.60	.43	.83	**.002**	.60	.43	.83	**.003**
Asian American	.24	.20	.28	**<0.001**	.24	.21	.29	**< .0001**
Latino/a	.27	.25	.29	**<0.001**	.27	.25	.29	**< .0001**
Other	1.07	.96	1.20	.209	1.08	.96	1.20	.188
White	Ref							
Age								
15–17	5.50	3.19	9.48	**< .0001**	5.54	3.22	9.54	**< .0001**
18–25	.53	.51	.56	**< .0001**	.53	.51	.55	**< .0001**
26–44	Ref							
Marital Status								
Divorced or Separated	2.01	1.90	2.12	**< .0001**	2.00	1.90	2.11	**< .0001**
Never Married	1.62	1.54	1.70	**< .0001**	1.62	1.55	1.71	**< .0001**
Widowed	.87	.79	.96	**.004**	.87	.79	.96	**.005**
Married	Ref							
Employment								
Unemployed	1.24	1.16	1.32	**< .0001**	1.24	1.16	1.32	**< .0001**
Full Time	Ref							
Major Depressive Episode Past Year								
Yes	1.62	1.52	1.72	**< .0001**	1.62	1.52	1.72	**< .0001**
No	Ref							
Substance Abuse								
Yes	5.60	5.18	6.04	**< .0001**	5.62	5.20	6.07	**< .0001**
No	Ref							
Health Insurance Coverage								
Yes	Ref							
No	1.57	1.50	1.64	**< .0001**	1.57	1.50	1.64	**< .0001**

*Note*. Data are from the National Survey on Drug Use and Health (NSDUH), 2007–2016. Sample sizes: rural non-pregnant women (n = 38,228), urban non-pregnant women, (n = 151,826), rural pregnant women (n = 1,938), and urban pregnant women (n = 6,424).

A second adjusted model also reported in Table 2 examined odds of smoking by geographic residence and pregnancy status to assess whether (a) the rural-urban differences reported in Fig 2 remained significant after adjusting for other smoking risk factors and (b) whether differences in smoking prevalence between non-pregnant versus pregnant women suggestive of pregnancy-related quitting were greater in urban than rural women, There was a significant interaction between residence and pregnancy status (*F* (2, 109) = 21.55, *p* < .0001). Adjusted odds of being a current smoker were consistently lower among urban compared to rural non-pregnant women (AOR = .80, [.77 –.85]) as well as urban compared to rural pregnant women (AOR = .62, [.50 –.76]) consistent with the differences shown in Fig 2. Additionally, while the odds of being a current smoker decreased by 25% among rural pregnant compared to non-pregnant women (AOR = .75, [.62 –.92]), the odds of smoking among urban pregnant compared to non-pregnant women decreased by 42% (AOR = .58, [.53 –.63]), suggesting greater pregnancy-related quitting among urban women. Adjusted odds of smoking in the overall sample also differed significantly across each of the selected covariates (see Table 2).

### Rural-urban comparisons on prevalence of nicotine dependence among current smokers

#### Overall prevalence

Prevalence of nicotine dependence, among current smokers only, for the overall samples of rural and urban smokers across the 10-year period was greater among rural than urban women overall throughout (*χ*^*2*^ (2) = 790.42, *p* < .0001). Prevalence of nicotine dependence was significantly greater among rural compared to urban women in 2007–2008 (*χ*^*2*^ (2) = 170.03, *p* < .0001), 2009–2010 (*χ*^*2*^ (2) = 148.77, *p <* .0001), 2011–2012 (*χ*^*2*^ (2) = 154.76, *p* < .0001), 2013–2014 (*χ*^*2*^ (2) = 167.98, *p* < .0001), and 2015–2016, (*χ*^*2*^ (2) = 209.40, *p* < .0001). Prevalence of nicotine dependence decreased over time among both rural (*χ*^*2*^ (8) = 31.66, *p* < .0001) and urban women (*χ*^*2*^ (8) = 212.79, *p* < .0001), but remained higher among rural compared to urban women throughout.

#### Prevalence by pregnancy status

Overall nicotine dependence across the ten-year period was higher among non-pregnant rural than urban women (*χ*^*2*^ (2) = 790.58, *p* < .0001). Prevalence of nicotine dependence was significantly greater among rural compared to urban women in 2007–2008 (*χ*^*2*^ (2) = 156.86, *p* < .0001), 2009–2010 (*χ*^*2*^ (2) = 143.83, *p <* .0001), 2011–2012 (*χ*^*2*^ (2) = 162.82, *p* < .0001), 2013–2014 (*χ*^*2*^ (2) = 157.45, *p* < .0001), and 2015–2016, (*χ*^*2*^ (2) = 215.19, *p* < .0001). Prevalence decreased over time among non-pregnant rural (*χ*^*2*^ (8) = 29.67, *p* = .0002) and urban women (*χ*^*2*^ (8) = 210.33 *p* < .0001) but remained greater among rural women throughout.

Overall nicotine dependence among rural pregnant women was significantly greater than urban pregnant women across the 10-year period (*χ*^*2*^ (2) = 63.69, *p* < .0001). Rural pregnant women demonstrated significantly higher nicotine dependence than urban pregnant women in 2007–2008 (*χ*^*2*^ (2) = 28.51, *p* < .0001), 2009–2010 (*χ*^*2*^ (2) = 21.00, *p <* .0001), 2013–2014 (*χ*^*2*^ (2) = 16.45, *p* = .0003), and 2015–2016, (*χ*^*2*^ (2) = 17.64, *p* = .0001), but not 2011–2012 (*χ*^*2*^ (2) = 1.86, *p* = .40). Neither rural (*χ*^*2*^ (8) = 13.79, *p* = .09) nor urban pregnant women (*χ*^*2*^ (8) = 10.41, *p* = .24) showed significant decreases in nicotine dependence over time.

#### Multivariable modeling of nicotine dependence

Table 3 shows results of the multivariable logistic regression models. Even after adjusting each of the selected smoking risk factors, nicotine dependence was greater among rural than urban women (AOR = 1.26, [1.19–1.34]). Pregnancy status also remained a significant, independent predictor of nicotine dependence in the adjusted models in that odds of nicotine dependence were greater among pregnant versus non-pregnant women (AOR = 1.25, [1.07–1.46]). There was no significant interaction between residence and pregnancy status. Odds of nicotine dependence also differed significantly across each of the selected covariates (see Table 3).

**Table 3:** Adjusted multivariable logistic regression coefficients for a model of nicotine dependence (in current smokers only) in the US between the years 2007–2016.

	Adjusted Model			
	AOR	95% CI		*p*
Rural Residence				
No	Ref			
Yes	1.26	1.19	1.34	**< .001**
Pregnancy Status				
No	Ref			
Yes	1.25	1.07	1.46	**.005**
Education				
12-17-year-olds	.43	.14	1.36	.15
< High School	3.21	2.88	3.59	**< .001**
High School Graduate	2.43	2.19	2.71	**< .001**
Some College	1.74	1.56	1.95	**< .001**
College Graduate	Ref			**< .001**
Income				**< .001**
Less than $20,000	1.55	1.42	1.70	**< .001**
$20,000 to $49,999	1.33	1.22	1.45	**< .001**
$50,000 to $74,999	1.19	1.09	1.31	**< .001**
≥ $75,000	Ref			
Race/Ethnicity				
African American	.86	.78	.94	**.001**
Native American	.54	.43	.66	**< .001**
Native Hawaiian / Pacific Islander	.79	.42	1.48	.45
Asian American	.51	.37	.70	**< .001**
Latino/a	.36	.32	.40	**< .001**
Other	.86	.72	1.03	.09
White	Ref			
Age				
15–17	1.52	.51	4.51	.45
18–25	.51	.48	.55	**< .001**
26–44	Ref			
Marital Status				
Divorced or Separated	1.03	.95	1.12	.47
Never Married	.88	.81	.95	**.001**
Widowed	.89	.74	1.05	.16
Married	Ref			
Employment				
Unemployed	1.32	1.19	1.47	**< .001**
Full Time	Ref			
Major Depressive Episode Past Year				
No	Ref			
Yes	1.46	1.35	1.57	**< .001**
Substance Abuse				
No	Ref			
Yes	1.61	1.45	1.78	**< .001**

*Note*. Data are from the National Survey on Drug Use and Health (NSDUH), 2007–2016. Sample sizes: rural non-pregnant women (n = 9,995), urban non-pregnant women, (n = 28,549), rural pregnant women (n = 489), and urban pregnant women (n = 1,014)

## Discussion

The overarching aim of the present study was to examine whether rural-urban disparities are impacting women of reproductive age (ages 15–44), including pregnant women. The current findings replicate earlier observations [5–6] that smoking prevalence is greater in rural than urban settings, differences that are apparent for both men and women [5] and extend these observations to women of reproductive age overall, and among rural non-pregnant and rural pregnant women examined separately. The results also demonstrate larger nonpregnant-to-pregnant reductions in odds of smoking (~17%) in urban compared to rural women, potentially suggesting a disparity in pregnancy-related smoking cessation. Smoking during pregnancy, the leading cause of poor pregnancy outcomes in the U.S. [16–17, 30], is already overrepresented among economically disadvantaged women and a major contributor to health disparities [28, 30–31]. Coupled with the risks of second-hand smoke exposure should these women parent young children [16–17], this may further increase such disparities with the potential for direct, multi-generational adverse health impacts.

In addition to greater smoking prevalence among women in rural settings, the current study demonstrated that prevalence of nicotine dependence among smokers was greater among rural than urban women of reproductive age overall, and among rural non-pregnant and rural pregnant women examined separately. To our knowledge, the present study is the first to report rural-urban differences in nicotine dependence. As nicotine dependence is a robust predictor of difficulties quitting smoking [25–26], including among pregnant women [20–23], this observation adds still another element to the growing association between rural residence and vulnerability to smoking and its associated adverse health impacts.

The differences noted above in smoking prevalence and nicotine dependence remained significant even after adjusting for common psychosocial, socioeconomic, and demographic smoking risk factors, adding to the accumulating evidence underscoring rural residence as an independent smoking vulnerability [5–6, 8]. It appears that beyond socioeconomic and psychosocial contributors to smoking risk among women of reproductive age, living in rural settings increases the likelihood of accumulating a profile of intersecting risk factors that may be uniquely contributing to rural-urban disparities in smoking and associated adverse health impacts [32–33].

### Implications for tobacco control and regulatory science

The current findings add additional rationale for greater tobacco control and regulatory efforts to decrease smoking among rural residents. The unsettling but clear findings that these rural-urban smoking disparities are impacting pregnant women adds urgency to addressing this disparity. Potential disparities in accessing health-care services, including evidence-based smoking cessation programs [31, 32–39] merit careful review. As has been suggested previously regarding these rural-urban disparities [4–6, 8], tobacco control and regulatory efforts may not be reaching rural populations with the same scope or intensity as in urban populations. An inverse relationship between population density and the amount of tobacco control community resources available has been noted previously [39]. We know of no comparable evidence regarding tobacco regulatory resources, but that possibility merits review especially regarding vulnerable populations such as women of reproductive age and pregnant women. Given a more limited public health infrastructure among rural communities [40], a combination of federal and state resources is likely to be necessary to eliminate these urban-rural disparities through increased tobacco regulatory efforts, such as increased excise taxes [41], clean indoor air policies in workplaces and other public venues [15, 39, 42–44], and targeted public health messaging [35, 45–46], particularly to rural women of childbearing age and healthcare providers who care for them.

### Limitations

Potential limitations include the observational nature of the study, which cannot support causal inferences, and the cross-sectional nature of the NSDUH survey. Although reliable differences over time were observed between rural-urban women overall and among non-pregnant and pregnant women examined separately, pregnancy-related quit estimates are based on cross-sectional data rather than following the same individuals longitudinally. As such, estimates may represent factors in addition to pregnancy-related smoking cessation. The precision of these estimates may be enhanced by longitudinal data collection. Furthermore, the public use NSDUH data files limit users to a county-level, dichotomous measure of rurality, rather than a continuous measure. This measure of rurality lacks specific geographic identifiers. As such, the current observations regarding rural disparities may be further enhanced through follow-up research using a data set that allows examination of specific geographical identifiers to evaluate the potential impact of state or local policies controls on rural-urban differences in smoking risk.

## Conclusion

Rural women, including pregnant women, lag behind their urban counterparts in reducing smoking prevalence, exhibit greater nicotine dependence, and what appears to be lower odds of quitting smoking during pregnancy, disparities that have potential for direct, multi-generational adverse health impacts. These findings highlight the need for more effective national and local tobacco control and regulatory efforts to reduce smoking among rural women of reproductive age.
